# The role of natural products targeting macrophage polarization in sepsis-induced lung injury

**DOI:** 10.1186/s13020-025-01067-4

**Published:** 2025-02-05

**Authors:** Yake Li, Sinan Ai, Yuan Li, Wangyu Ye, Rui Li, Xiaolong Xu, Qingquan Liu

**Affiliations:** 1 Beijing Hospital of Traditional Chinese Medicine, Capital Medical University, Beijing, 100010 China; 2 Beijing Institute of Chinese Medicine, Beijing, 100010 China; 3 Laboratory for Clinical Medicine, Capital Medical University, Beijing, 100010 China; 4 China-Japan Friendship Hospital, Beijing, 100029 China; 5 Henan University of Chinese Medicine, Zhengzhou, 450046 China; 6 Guang’anmen Hospital, China Academy of Chinese Medical Sciences, Beijing, 100053 China

**Keywords:** Traditional Chinese herbs, Natural products, Sepsis-induced acute lung injury, SALI, Macrophage polarization, M1, M2

## Abstract

Sepsis-induced acute lung injury (SALI) is characterized by a dysregulated inflammatory and immune response. As a key component of the innate immune system, macrophages play a vital role in SALI, in which a macrophage phenotype imbalance caused by an increase in M1 macrophages or a decrease in M2 macrophages is common. Despite significant advances in SALI research, effective drug therapies are still lacking. Therefore, the development of new treatments for SALI is urgently needed. An increasing number of studies suggest that natural products (NPs) can alleviate SALI by modulating macrophage polarization through various targets and pathways. This review examines the regulatory mechanisms of macrophage polarization and their involvement in the progression of SALI. It highlights how NPs mitigate macrophage imbalances to alleviate SALI, focusing on key signaling pathways such as PI3K/AKT, TLR4/NF-κB, JAK/STAT, IRF, HIF, NRF2, HMGB1, TREM2, PKM2, and exosome-mediated signaling. NPs influencing macrophage polarization are classified into five groups: terpenoids, polyphenols, alkaloids, flavonoids, and others. This work provides valuable insights into the therapeutic potential of NPs in targeting macrophage polarization to treat SALI.

**Graphical Abstract:**

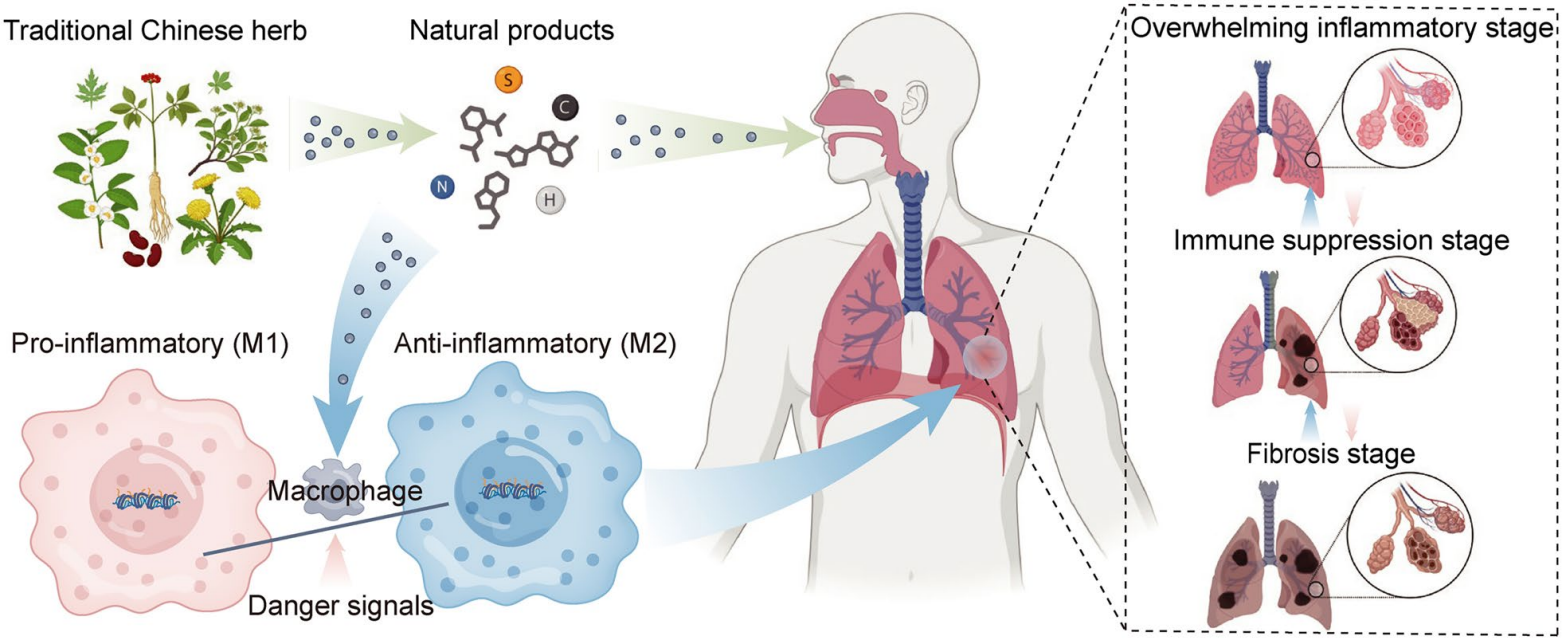

## Highlights

- This paper reviews the characteristics of macrophage polarization and explores the signaling pathways that regulate polarization phenotypes.
- This paper reviews the changes in macrophage phenotypes and their regulatory mechanisms during the three stages of sepsis-induced lung injury: the overwhelming inflammatory phase, the immunosuppressive phase, and the fibrotic phase.
- This paper reviews natural compounds with potential therapeutic effects on macrophage polarization-related sepsis-associated lung injury (SALI) and explores their mechanisms of action against SALI.

## Background

Sepsis-induced acute lung injury (SALI) is a major global medical challenge with a high mortality rate and poses a severe threat to public health [1, 2]. During the progression of SALI, pathogens such as bacteria and endotoxins invade lung tissue, triggering the release of inflammatory cytokines and activating alveolar macrophages, epithelial cells, T cells, B cells, neutrophils, and other immune cells [2–4]. This process initiates an uncontrolled inflammatory response, leading to severe lung damage and impaired function.

Macrophage polarization plays a crucial role in the pathogenesis of SALI [5, 6]. As the primary immune cells in the lungs, macrophages are responsible for recognizing airborne pathogens and initiating innate immune responses, playing a central role in the SALI inflammatory. Macrophages exhibit remarkable plasticity, allowing them to polarize into either the M1 or M2 phenotype in response to different microenvironmental signals [7]. Additionally, the polarization state of macrophages plays a pivotal role in the development of pulmonary inflammation and fibrosis at different stages of SALI [8]. During the initial inflammatory phase of sepsis, M1 macrophages release cytokines such as tumor necrosis factor-alpha (TNF-α) and interleukin-6 (IL-6), intensifying lung injury [9]. IL-6 levels in serum typically range from 1 to 25 pg/mL, but can exceed 1 ng/mL in sepsis. As an early mediator of inflammation, IL-6 promotes T and B lymphocyte proliferation, and stimulates acute-phase protein synthesis, peaking within 2 h of inflammation onset. IL-6 responds faster than CRP and PCT to infection, making it a key clinical biomarker [10]. In contrast, M2 macrophages release anti-inflammatory factors such as IL-10, mitigating inflammation [11]. During the immunosuppressive phase, macrophages tend to polarize to the M2 phenotype, which may lead to immune suppression, increasing the risk of secondary infections. IL-10, an anti-inflammatory cytokine secreted by M2 macrophages, is inversely correlated with prognosis in sepsis—higher levels of IL-10 are linked to worse outcomes. In studies, LPS stimulation of PBMCs from septic patients results in significantly lower TNF-α levels compared to non-septic patients. Sepsis patients with TNF-α production below 200 ng/L in LPS-stimulated monocytes are considered to be in an immunosuppressive state [12]. During the late fibrotic phase, macrophages can promote fibrosis by producing growth factors, such as transforming growth factor-beta (TGF-β) and CCL18, but they may also inhibit fibrosis by producing matrix metalloproteinases (MMPs). In pulmonary fibrosis, serum levels of CCL18 are significantly higher in patients compared to healthy controls. Clinical studies have demonstrated that CCL18 serves as an important biomarker for assessing the progression of pulmonary fibrosis, with elevated levels closely associated with disease severity [13]. Recent clinical studies have revealed that granulocyte-macrophage colony-stimulating factor can improve the prognosis of patients with sepsis-induced immune paralysis and effectively reduce secondary infections. This underscores the significant clinical value of further exploring therapeutic agents targeting macrophage polarization [14]. Therefore, studying the regulatory mechanisms of macrophage polarization represents a significant avenue for developing new therapies for SALI.

Recently, accumulating evidence has highlighted the potential use of natural products (NPs) in treating SALI. A variety of novel NPs isolated from, plants and other organisms have enriched chemical libraries and shown anti-inflammatory potential. Compared with conventional synthetic molecules, NPs have unique properties that have both advantages and disadvantages for the drug discovery process. In general, NPs have higher molecular masses, more sp3 carbon and oxygen atoms but fewer nitrogen and halogen atoms, more H-bond acceptors and donors, lower octanol-water partition coefficients (CLogP values, which indicate higher hydrophilicity), and higher molecular stiffness than synthetic compounds [15]. NPs, including flavonoids, alkaloids, and terpenoids, inhibit the proinflammatory response of M1-type macrophages or promote the reparative effects of M2-type macrophages, resulting in anti-inflammatory activity and alleviating lung injury, particularly in in vitro and in vivo SALI treatments. Nevertheless, there is a dearth of comprehensive syntheses of NPs for macrophage polarization toward the treatment of SALI. This work aims to address this gap in the literature by providing a thorough analysis of the effects of NPs on macrophage polarization and the specific mechanisms involved in treating SALI. Therefore, this paper critically reviewed the relevant data in the PubMed and Web of Science databases from 2017 to 2024 (up to December). The search terms included sepsis-induced acute lung injury, macrophage polarization and natural products. Taken together, this paper reviews the natural compounds in the available literature regarding their protective effects against SALI and their underlying mechanisms. We hope that this review will serve as a valuable reference and guide for future research and clinical applications.

## Macrophage polarization and major regulatory signaling pathways

M1 macrophages are proinflammatory, thus aggravating inflammatory responses, whereas M2 macrophages are anti-inflammatory, thus inhibiting M1 macrophages, clearing apoptotic cells, and promoting tissue repair [16]. M1 macrophages polarize proinflammatory signals such as interferon-gamma (IFN-γ) and lipopolysaccharide (LPS) [17, 18] stimulating the release of various inflammatory cytokines, including TNF-α, interleukin-1 beta (IL-1β), IL-12, IL-6, IL-23, macrophage inflammatory protein-2 (MIP-2), monocyte chemoattractant protein-1 (MCP-1), cyclooxygenase-2 (COX-2), reactive oxygen species (ROS), and nitric oxide (NO). M1 macrophages play a crucial role in pathogen clearance, promoting inflammatory responses, and activating T cells. However, sustained activation of M1 polarization is connected to the progression and growth of diverse inflammatory and autoimmune diseases [19]. M2 macrophages are associated with anti-inflammatory and tissue repair processes [20]. They typically polarize in response to anti-inflammatory cytokines, such as interleukin-4 (IL-4) and IL-13 [21]. M2 macrophages can release anti-inflammatory molecules such as TGF-β, arginase-1 (Arg-1), and IL-10, which help alleviate inflammation, promote tissue repair, and suppress the activity of effector T cells [22]. M2 macrophages are vital for controlling immune reactions, promoting tissue remodeling, preventing immune hyperactivation, and maintaining immune homeostasis [23]. However, excessive responses can also lead to immune paralysis in the host [24].

**Fig. 1:**
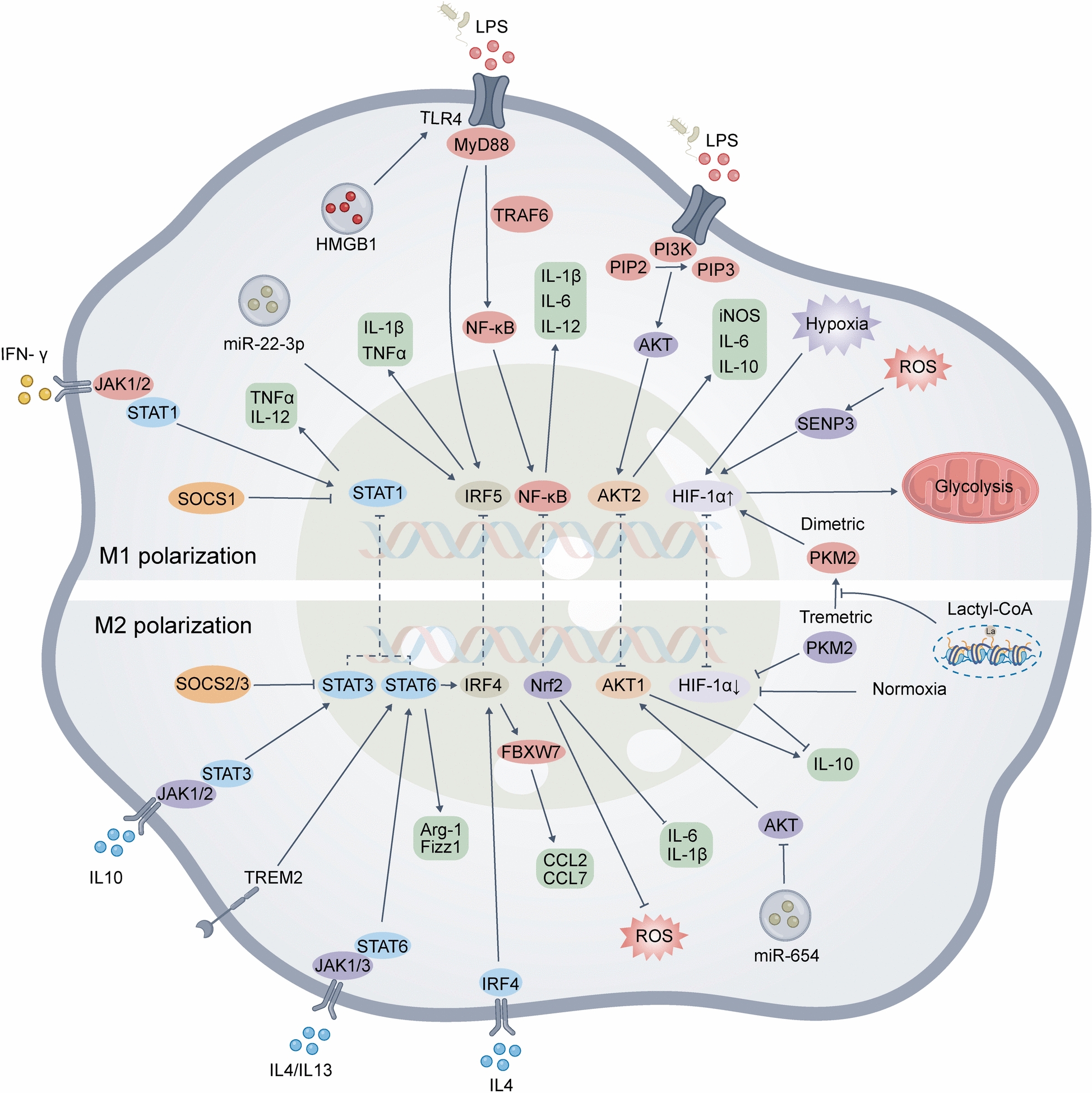
Macrophage polarization is a key regulatory process in the immune response. The schematic depicts the presence of both M1 and M2 polarization processes in the cell, with the upper half representing proinflammatory M1 phenotype and the lower half representing anti-inflammatory M2 phenotype, and the figure depicts in detail the involvement of multiple intracellular signaling pathways in the regulation of this complex polarization mechanism, revealing the molecular-level interactions involved in the macrophage polarization process

The polarization state of macrophages has profound implications for host immune homeostasis and disease progression [25–27]. During severe sepsis, having a balance between the M1 and M2 phenotypes is crucial for controlling inflammatory responses, preventing immune suppression, and promoting recovery. Increasing evidence suggests that modulating the macrophage polarization status could be a potential tactic for treating sepsis [28–30]. We summarized the phenotype and function of macrophages stimulated by different factors and the exact mechanism comprising signaling pathways (Fig. 1).

### Phosphoinositide 3-kinase (PI3K)/protein kinase B (AKT) signaling pathways

Phosphoinositide 3-kinase (PI3K) is a dimeric complex composed of catalytic and regulatory subunits. It phosphorylates phosphatidylinositol-4,5-bisphosphate (PIP2) into phosphatidylinositol-3,4,5-trisphosphate (PIP3), promoting the activation of downstream AKT (protein kinase B) [31]. The PI3K/AKT pathway is closely linked to macrophage polarization [32]. Li and colleagues reported that inhibiting PI3K/AKT in macrophages suppresses M2 polarization [33]. The regulation of macrophage activation by PI3K/AKT is mediated primarily through AKT and its downstream molecules. AKT1 is associated with M1 polarization, and in its absence, macrophages show increased inducible nitric oxide synthase (iNOS), IL-6, and TNF-α expression, promoting M1 polarization. Conversely, loss of AKT2 leads to elevated levels of Fizz1, Chi3l3, and IL-10 in macrophages, promoting M2 polarization [34–36]. Downstream molecules such as mTOR and TSC also play roles in regulating macrophage polarization. TSC2 functions as a suppressor of mTOR signaling. Interference with TSC2 using siRNA promotes mTOR activation and increases the expression of STAT3 and IL-10, thereby promoting M2 polarization [37], whereas rapamycin inhibition of mTOR drives macrophages toward M1 polarization [38]. Recent studies have shown that IL-10 rapidly activates macrophage PI3K and downstream AKT, thereby activating mTORC1. However, mTORC1 activation may inhibit AKT1 activity, leading to reduced M2 polarization [39]. The PI3K/AKT pathway plays a crucial role in regulating macrophage polarization by modulating the balance between M1 and M2 phenotypes. Activation of PI3K promotes the activation of the AKT family, with AKT1 driving M2 polarization and AKT2 favoring M1 polarization. mTORC1 activation supports M2 polarization, while inhibition of mTOR by rapamycin shifts macrophages towards M1 polarization.

### TLR4/NF-kB signaling pathways

Upon being activated by pathogen-associated molecular patterns such as LPS, Toll-like receptor 4 (TLR4) triggers NF-κB activation, leading to the release of inflammatory cytokines associated with M1 macrophages [40]. TLR4 triggers NF-κB activation through both MyD88-dependent and MyD88-independent pathways [41]. In the MyD88-dependent pathway, the intracellular Toll/interleukin-1 receptor (TIR) domain of TLR4 binds to the carboxyl terminus of MyD88, activating TRAF6, leading to the phosphorylation of NF-κB inhibitor (IκB) and the release of NF-κB, thus increasing the expression of M1-associated factors such as IL-18, IL-6, and IL-12 [42–44]. TRAF6 participates in the regulation of M1/M2 polarization and the induction of related genes. Research by Wen et al. revealed that upregulating TRAF6 downregulates the expression levels of the M2-related genes CD206 and Arg-1, thereby inhibiting M2 polarization and alleviating allergic inflammation [45]. However, other studies have reported that upregulating TRAF6 promotes the expression of M1 macrophage-related genes such as CD11c, and iNOS, and proinflammatory cytokines, including TNF-α, IL-1β, and IL-6, accelerating the progression of diabetic peripheral neuropathy [46]. When the MyD88-independent pathway is activated, interferon regulatory factor 3 (IRF3) is activated and translocates into the cell nucleus, thereby inducing the secretion of type I interferons (IFNs), which are associated with M1 polarization [47–49]. Upon activation by LPS, TLR4 triggers NF-κB activation via both MyD88-dependent and MyD88-independent pathways. In the MyD88-dependent pathway, TLR4 activates TRAF6, promoting M1 polarization through upregulation of IL-6 and TNF-α. In the MyD88-independent pathway, IRF3 activation induces type I interferon secretion, further driving M1 polarization.

### JAK/STAT signaling pathways

JAKs (Janus kinases) are a type of non-receptor tyrosine kinase that participate in signal transduction upon binding with cytokine receptors [50]. Mammals contain four members of the JAK family: JAK1, JAK2, JAK3, and Tyk2 [51] Signal transducers and activators of transcription (STATs) are a group of transcription factors capable of binding to DNA [52]. In their normal state, STATs are located in the cytoplasm, but once activated, they form dimers, translocate to the cell nucleus, and participate in the control of gene expression. The JAK/STAT signaling pathway plays a crucial role in regulating macrophage polarization [53]. Activated STAT1 is closely linked to the differentiation and activation of M1 macrophages, promoting the expression of proinflammatory cytokines such as IL-12 and TNF-α upon their translocation to the cell nucleus [54]. However, STAT6 serves as a central regulatory factor in M2 polarization [55], playing a critical role in IL-4 and IL-13 signaling. Upon binding with their receptors, IL-4 and IL-13 activate the associated JAK kinases, leading to the phosphorylation and activation of STAT6. Activated STAT6 then translocates into the nucleus, where it promotes the expression of genes associated with M2 macrophages, such as Arg1 and Fizz1, which play a crucial role in anti-inflammatory responses and tissue repair [56]. Suppressor of cytokine signaling (SOCS) proteins are target gene products activated by STAT proteins that can negatively regulate the JAK/STAT pathway, thereby influencing M1/M2 polarization [57]. Li et al. reported that overexpressing SOCS2 inhibits M2 macrophage polarization and reduces the proliferation and migration of cervical cancer cells by suppressing the IL-6/JAK2/STAT3 pathway [58]. Shi et al. reported that the activation of the JAK2/STAT3/SOCS3 signaling pathway promotes the polarization of macrophages towards the proinflammatory M1 subtype [59]. Liang et al. reported that downregulation of SOCS1 promotes the M1 macrophage phenotype via the JAK1/STAT1 pathway [60]. In conclusion, the JAK/STAT pathway regulates macrophage polarization through STAT1 and STAT6 activation. STAT1, activated by JAK1/JAK2, promotes M1 polarization via IL-12 and TNF-α, while STAT6, activated by IL-4/IL-13 through JAK1/JAK3, drives M2 polarization via Arg1 and Fizz1. SOCS proteins, activated by STATs, modulate polarization, with SOCS2 inhibiting M2 and SOCS3 promoting M1.

### IRF related signaling pathways

IRFs are transcription factors that play crucial roles in regulating macrophage polarization [61]. IRF4 regulates the activation of M2 macrophages, a process that is associated with the histone demethylase JMJD3 [62]. Research has shown that IRF4 expression is suppressed in LPS-induced ALI models. Increasing IRF4 expression levels can activate the miR-132-3p/FBXW7 axis, thereby promoting M2 macrophage polarization and alleviating lung injury [63]. However, Wang et al. reported that IRF4 promotes cell apoptosis and exacerbates inflammation in an ulcerative colitis model through the Bcl6/JAK2/STAT3 pathway, promoting M1 polarization of macrophages [64]. Conversely, the activation of IRF5 is associated with the proinflammatory function of macrophages [65]. IRF5 activates signaling pathways such as MyD88 and TLRs, promoting M1 polarization of macrophages and inhibiting the activation of M2 macrophages [66]. Liang et al. reported that IRF5 silencing shifts the polarization state of rat peritoneal macrophages from M1 to M2 in rats with depressive colitis and non-depressive colitis, in which the shift is more pronounced, accompanied by a downregulation of TNF-α and IL-1β expression levels [67, 68]. Furthermore, increasing miR-22-3p inhibits IRF5, promotes macrophage polarization toward the M2 type and suppresses inflammation in tissues, thus alleviating the severity of spinal cord ischemia/reperfusion injury [69]. In summary, IRFs, such as IRF4, promote M2 polarization through the miR-132-3p/FBXW7 axis to alleviate lung injury but can also drive M1 polarization via the Bcl6/JAK2/STAT3 pathway. IRF5 promotes M1 polarization by activating MyD88 and TLR pathways while inhibiting M2 activation, whereas miR-22-3p inhibits IRF5, shifting macrophages toward the M2 phenotype.

### Hypoxia-inducible factor-1 (HIF) related signaling pathways

Hypoxia-inducible factor-1 (HIF-1) is a transcription activator that responds to hypoxia and is involved in regulating both acute and chronic inflammation [70, 71] Under hypoxic conditions, HIF-1 stimulates glucose uptake, the expression of key glycolytic enzymes, and pyruvate dehydrogenase kinase-1, thereby promoting metabolic reprogramming that results in M1 macrophage polarization [72]. HIF-1α promotes M1 macrophage polarization, potentially by enhancing glycolytic metabolism [73]. Recent studies have shown that the ubiquitination and subsequent degradation of HIF-1α through the proteasome pathway inhibits glycolysis and prevents M1 macrophage polarization [74]. SUMO-specific peptidase 3 (SENP3) is a deSUMOylating enzyme that is abundantly produced in response to ROS during acute lung injury (ALI) [75]. Recent studies have shown that SENP3 enhances M1 macrophage polarization and the production of proinflammatory cytokines via the PKM2/HIF-1α axis, which contributes to lung injury [76]. Another study indicated that HIF-1α is associated with M2 macrophage polarization. Allogeneic adipose-derived stem cells can induce M2 macrophage polarization through the HIF-1α/IL-10 pathway, thereby promoting the repair of ischemic muscles [77]. In summary, HIF-1 plays a multifaceted role in macrophage polarization, with HIF-1α influencing both M1 and M2 polarization through metabolic reprogramming and various signaling pathways, which has implications for both inflammation and tissue repair.

### NRF2 related signaling pathways

Nuclear factor erythroid 2-related factor 2 (NRF2) is a pivotal transcription factor involved in the cellular antioxidant response that influences macrophage polarization [78]. In their normal state, NRF2 protein levels are typically low because of regulation by three E3 ubiquitin ligase complexes. The primary regulatory mechanism is KEAP1-CUL3-RBX1, followed by β-TrCP-SKP1-CUL1-RBX1 and HRD1, which mediate the ubiquitination and subsequent proteasomal degradation of NRF2 [79]. NRF2 is suggested to play a pivotal role in regulating proinflammatory cytokine expression and intracellular ROS levels. In an M1-type macrophage inflammation model, NRF2 exerts an anti-inflammatory effect by directly scavenging ROS and inhibiting M1 polarization by blocking the transcription of the IL-6 and IL-1β genes [80]. Furthermore, NRF2 overexpression promotes PPARγ expression in the nucleus and inhibits the LPS-induced nuclear translocation of NF-κB, thereby suppressing M1 polarization [81]. STUB1 (also known as CHIP) is a chaperone-dependent E3 ubiquitin ligase, and recent studies have demonstrated its involvement in regulating macrophage polarization. The silencing of STUB1 inhibits NRF2 ubiquitination, promoting M2 macrophage polarization and attenuating the inflammatory response [82]. AMPK also activates NRF2/heme oxygenase 1 (HO-1), exerts anti-inflammatory effects and promotes macrophage polarization toward the anti-inflammatory M2 phenotype [83, 84]. In brief, NRF2 suppresses M1 polarization by scavenging ROS, inhibiting IL-6 and IL-1β transcription, and blocking NF-κB activation. Silencing STUB1 stabilizes NRF2, facilitating M2 polarization, while AMPK activation of the NRF2/HO-1 pathway further promotes the anti-inflammatory M2 phenotype.

### *High mobility group protein box 1 (HMGB1) related signaling pathways*

HMGB1 is a multifunctional protein crucial to the innate immune system, especially in response to tissue injury and infection [85]. HMGB1 exerts a bidirectional regulatory influence on macrophage polarization and affects both M1 and M2 macrophages [86]. During M1 polarization, HMGB1 promotes inflammatory responses and cytokine production by binding to receptors such as the receptor for advanced glycosylation end products (RAGE) and TLR4 [87]. Moreover, HMGB1 can selectively inhibit TLR4 signaling through interactions with molecules such as CD24, contributing to M2 polarization and anti-inflammatory responses [88]. Recent studies have shown that HMGB1 activates the absent in melanoma 2 (AIM2) inflammasome in macrophages and induces M1 polarization via TLR2, TLR4, and the RAGE/NF-κB signaling pathway [89]. Li et al. reported that treating with recombinant human HMGB1 significantly increased the level of HMGB1 in supernatants from THP-1 cell line-derived macrophages [90]. These findings suggest that HMGB1 is a key mediator of macrophage polarization and inflammatory responses. In short, HMGB1 promotes M1 polarization through the TLR2, TLR4, and RAGE/NF-κB pathways, while facilitating M2 polarization by selectively inhibiting TLR4 signaling via CD24. This dual role underscores its importance in balancing immune responses.

### Triggering receptor expressed on myeloid cells 2 (TREM2) related signaling pathways

TREM2 is an immunomodulatory receptor located on the surface of myeloid cells. It plays a crucial role in mediating immune responses as a signaling hub [91]. TREM2 regulates macrophage phagocytosis and inflammatory responses [92]. Additionally, it promotes macrophage polarization, facilitating the transition from the M1 phenotype to the M2 phenotype [93] Elevated TREM2 expression impedes NF-κB signaling activation, reducing proinflammatory cytokine release and facilitating M2 polarization [94]. In TREM2 knockout mice, the lung inflammatory response is intensified following bacterial infection, which is linked to suppressed M2 polarization. However, recent findings suggest that TREM2 may have bidirectional roles in various pathological states [95]. In a pulmonary fibrosis mouse model, TREM2 activation promoted STAT6 signaling, driving M2 polarization. This process leads to the production of profibrotic factors, such as platelet-derived growth factor (PDGF) and Fizz1, which exacerbate pulmonary fibrosis [96]. Moreover, in a kidney injury model, TREM2 deficiency facilitates M1 polarization via enhanced STAT1 signaling while promoting M2 polarization through the augmented STAT3 signaling pathway [97]. These findings suggest that TREM2 has a multifaceted role in regulating macrophage polarization and inflammatory responses, with specific effects depending on the biological context and pathological state. In conclusion, TREM2 promotes M2 macrophage polarization by inhibiting NF-κB and activating STAT6 or STAT3 pathways, while suppressing M1 polarization through STAT1 inactivation. This regulation reduces inflammation but may exacerbate conditions like pulmonary fibrosis by driving the production of profibrotic factors.

### PKM2-related signaling pathways

PKM2, a member of the pyruvate kinase (PK) family, is a pivotal regulatory enzyme in glycolysis and plays a vital role in metabolic regulation, particularly in the Warburg effect [98]. PKM2 acts as a metabolic switch in macrophage glucose metabolism, with its tetrameric form exhibiting high PK activity and its dimeric form functioning primarily as a protein kinase that stabilizes HIF-1α [76]. The HIF-1α/PKM2 axis is crucial for macrophage polarization and lung injury [99]. PKM2 inhibits the activation of LPS-induced M1 macrophages while promoting M2 polarization and increasing CD206 expression. This process involves inhibiting PKM2 dimer and tetramer formation and hindering aerobic glycolysis reprogramming [100]. Recent studies have revealed that lactic acid inhibits inflammatory metabolic adaptation in proinflammatory M1 macrophages by increasing PKM2 lactylation and inhibiting its transition from tetramers to dimers [101]. These findings confirm the regulatory role of PKM2 in inflammatory metabolic adaptations within macrophages. In summary, the dimeric form of PKM2 promotes M1 polarization by stabilizing HIF-1α and enhancing glycolysis, while its tetrameric form drives M2 polarization by increasing CD206 expression. Lactic acid enhances PKM2 lactylation, preventing the transition from the tetrameric form to the dimeric form.

#### Exosome

Exosomes play an important role in intercellular communication, and they are capable of carrying mRNA, DNA, miRNA, proteins, lipids, and many other substances [102]. It has been found that a variety of exosome-like vesicles of plant origin have shown potential therapeutic effects on anti-inflammatory activity and oxidative stress in in vitro and in vivo experiments [103]. Nevertheless, the ability of exosomes to directly regulate inflammatory cells has not been extensively studied, and they indirectly regulate gene expression mainly by delivering functional miRNAs to immune cells. miRNAs are a class of small non-coding RNA molecules that regulate gene expression at the post-transcriptional level by binding to the 3'UTR region of target genes [104]. A growing number of studies have revealed a close link between miRNAs and macrophage polarization phenotypes [105]. The miRNAs associated with M1-type polarization include miR-34a, miR-125a, miR-125b, miR-146a, miR-155, miR-9, and miR-200c; whereas the miRNAs associated with M2-type polarization include miR-21, miR-124, miR-125a, miR-146a, miR-146b, miR-181b, miR-200c, miR-223, and miR-511[106]. miRNAs are able to participate directly in the M1/M2 polarization process. For example, miR-155 is thought to be a key molecule in promoting M1-type macrophage polarization, and its expression can be upregulated by IFN-β, TNF-α, IL-1, TLR2, TLR3, TLR4, and TLR9 [107]. Research revealed that the miR-155/SOCS1 axis is involved in the PI3K/Akt1-mediated S. aureus-induced respiratory infection mouse model on macrophage phenotype switching [108]. miR-21 promotes M2-type macrophage polarization, and mesenchymal stem cells (MSCs) secrete miR-21-rich exosomes under hypoxic conditions, which further induces M2 polarization and promotes the development of lung cancer [109]. Programmed cell death protein 4 (PDCD4) is a tumor suppressor that activates NF-κB and inhibits IL-10 expression. Recent studies have shown that miR-21 can negatively regulate PDCD4. miR-21-induced M2-type polarization was followed by significant improvement of symptoms and survival in a mouse model of sepsis [110]. In summary, exosomes carrying miRNAs like miR-155 promote M1 polarization, while miR-21 enhances M2 polarization, with MSC-derived exosomes under hypoxia further promoting M2 polarization.

There are potential interactions between different signaling pathways in the regulation of macrophage polarization. Activation of the PI3K/AKT signaling pathway can reduce the stimulation of macrophages by LPS, decreasing the activation of NF-κB and the expression of iNOS, thereby inhibiting the M1 macrophage response [111]. Additionally, inhibition of the PI3K/AKT pathway lowers the activation of the mTOR/HIF-1α axis and the associated glycolytic processes [112]. However, S-glutathionylation of PKM2 promotes the dissociation of PKM2 tetramers into inactive dimers, which translocate to the nucleus and activate HIF1α-dependent gene expression, while inducing glycolytic enzymes and pro-inflammatory proteins, thus reprogramming macrophages to a high-inflammatory phenotype [113]. Studies have shown that in NRF2 inhibition models, the activation of the NF-κB pathway is more pronounced, promoting the M1 inflammatory response [114]. NRF2 activation can suppress STAT3, thereby blocking the production of M1 pro-inflammatory cytokines and chemokines [115]. HMGB1 induces M1 macrophage polarization by activating the AIM2 inflammasome in macrophages and through TLR2, TLR4, and RAGE/NF-κB signaling pathways [116].

## Regulatory role of macrophage polarization in different stages of SALI

### Overwhelming inflammatory stage

**Fig. 2:**
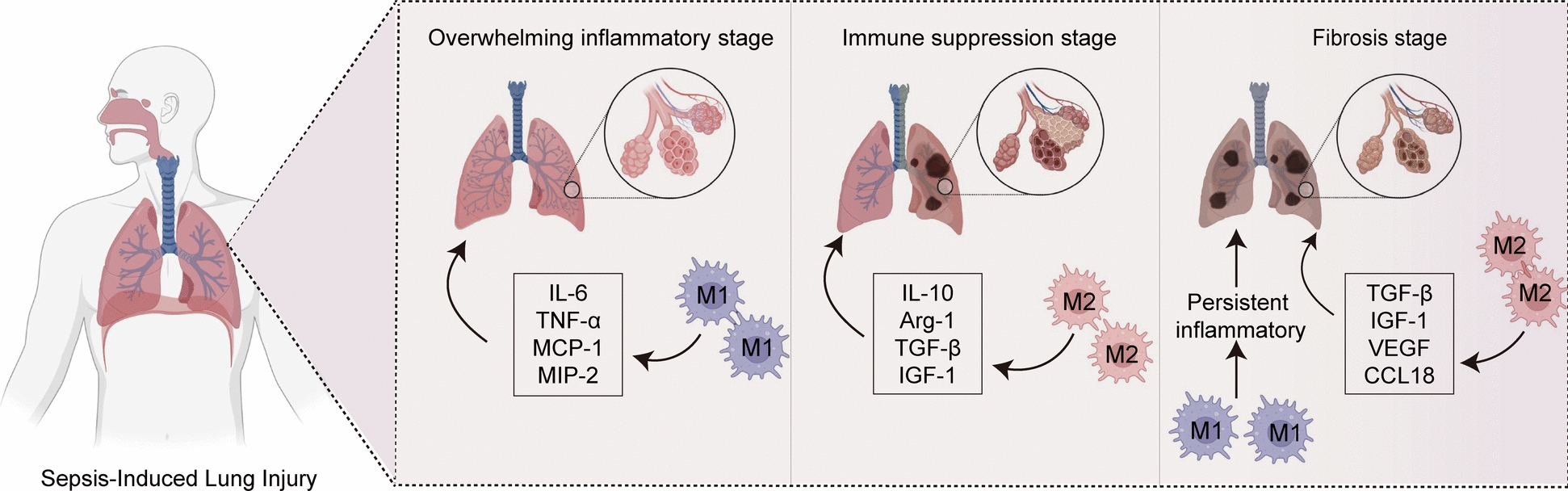
Potential molecular mechanisms of macrophage polarization during the three phases of SALI. The overwhelming inflammatory stage, the immune suppression stage, and the fibrosis stage, respectively. Among them, the overwhelming inflammatory stage was dominated by M1 phenotype activation, the immune suppression stage was dominated by M2 phenotype activation, and the fibrosis stage was associated with both M1 phenotype and M2 phenotype

Sepsis is a progressive response caused by infection, leading to overactivation of inflammatory cells, an imbalance between proinflammatory and anti-inflammatory mechanisms, and the initiation of an uncontrolled inflammatory response (Fig. 2). This excessive inflammation is a key factor in sepsis-induced organ dysfunction [117]. Inhibiting M1 macrophage polarization during this stage can significantly reduce proinflammatory factor release, alleviate organ damage and reduce patient mortality [118]. Clinical studies in ARDS patients also revealed that IFIH1, IRF1, STAT1, IFIT3, and GBP1 promote M1 polarization during the early stage [119–121]. In the LPS-or CLP-treated experimental models, SALI begins with macrophage polarization toward the M1 phenotype, with a marked inflammatory response occurring within 24 h [122, 123]. In LPS induced mouse models, miR-19a-3p targets STAT1, binding to its 3'UTR, thus inhibiting M1 polarization and alleviating lung inflammation [124]. Polymorphonuclear neutrophil (PMN)-derived exosomes promote inflammation in SALI by driving M1 polarization [125]. Studies have shown that miR-30d-5p in PMN-derived exosomes induces M1 polarization via NF-κB activation [5]. Similarly, the iHvKp-exo, isolated from RAW264.7 macrophages activated p38-MAPK and promoted M1 polarization. The miR-155-5p in exosomes further amplifies this effect by targeting the MSK1/p38-MAPK axis, exacerbating lung injury [126]. These findings suggest that targeting exosomes could be a potential therapeutic strategy for ALI. Additionally, inhibiting aerobic glycolysis has been identified as another strategy for suppressing M1 polarization. He et al. reported that WIN55212-2, a cannabinoid receptor agonist, inhibits macrophage glycolysis and M1 polarization, which suppresses FOXO3 expression [127]. Additionally, HIF-1α plays a crucial role in glycolytic metabolism by upregulating glycolytic enzyme expression [128]. SET domain-containing 2 (SETD2) is a methyltransferase that catalyzes H3K36me3, thereby suppressing HIF-1α expression in SALI models and inhibiting glycolysis and M1 polarization [129]. Further studies suggest that enhancing M2 polarization in sepsis can also reduce inflammatory factor secretion and improve survival rates [130]. Tang et al. reported that soluble egg antigens produced during Japanese schistosome infection stimulate M2 polarization by activating the STAT6 and PI3K pathways, improving survival in septic mice [131]. In the overwhelming inflammatory stage, M1 macrophage polarization predominates, significantly contributing to the inflammatory response and organ dysfunction. Pathways such as STAT1 and NF-κB, as well as the suppression of glycolysis via HIF-1α and SETD2, promote M1 polarization. Conversely, M2 polarization is inhibited during this stage, involving pathways such as STAT6 and PI3K.

### Immune suppression stage

Sepsis progresses to a late phase characterized by prolonged immune suppression [132], which is characterized by increased secondary infections [133], reduced inflammatory cytokine release [134], and an elevated risk of mortality. Research indicates that M2 macrophages play a critical role during the immune suppression phase of sepsis [135]. During this phase, macrophages polarize toward the M2 phenotype, releasing numerous anti-inflammatory factors such as IL-10. They also suppress the antigen presentation capability of macrophages by reducing the surface levels of HLA-DR [136], resulting in immune paralysis and an increased risk of secondary infections. Thus, modulating macrophage polarization to inhibit M2 or promote M1 polarization may be a potential therapy for addressing the immunosuppressive phase of sepsis. Extracellular cold-inducible RNA-binding protein (eCIRP) is a proinflammatory mediator that promotes endotoxin tolerance in macrophages [137]. During the hypodynamic phase of sepsis, there is a significant increase in eCIRP expression. Research findings indicate that eCIRP can activate STAT3 by binding to the IL-6R receptor, thereby promoting M2 polarization and leading to immune tolerance [138]. By blocking the binding of eCIRP to IL-6R, macrophages release more proinflammatory mediators (such as TNF-α), leading to an increase in M1 polarization and improved immune tolerance [138]. Liu et al. reported that overexpressing ATF4, an important glycolysis activator, can ameliorate the reduced levels of lactate secretion, proinflammatory cytokines, and HK2 expression in LPS-induced tolerant macrophages, thereby improving the immune tolerance of macrophages in sepsis [139, 140]. Furthermore, enhancing glycolysis is critical for the proinflammatory response of macrophages during sepsis-induced immunosuppression [141]. McBride et al. reported that when sepsis-induced immune dysfunction leads to secondary infections, treating with β-glucan or monophosphoryl lipid A can alter macrophage immunometabolism and mitochondrial function, increase glycolysis, and promote M1 polarization, thereby eliminating pathogens [142]. Transcriptomic and metabolic analyses of human sepsis patients have revealed that the transition from oxidative phosphorylation to aerobic glycolysis is crucial for activating the host's initial defense system [143, 144]. Certain miRNAs can also modulate macrophage tolerance and serve as indicators of immune dysfunction and unfavorable outcomes in individuals with sepsis [145]. In sepsis patients, increased expression of miR-221/222 is associated with increased immunoparalysis and organ damage. Seeley et al. demonstrated that the upregulation of miR-221/222 modulates Brahma-related gene 1 (Brg-1), resulting in the transcriptional suppression of genes involved in inflammation and worsening immunotolerance [136].

Immunosuppressive molecules are essential for regulating the immune cell inhibitory state, and play a significant role in the pathophysiology of sepsis [146]. T-cell immunoglobulin and mucin domain-containing protein 3 (Tim-3) is a crucial negative regulatory molecule [147], and the signaling pathway with galectin-9, plays a critical role in maintaining immune balance during sepsis. During the advanced phases of sepsis, Tim-3 expression decreases [148], hampering M2 macrophage polarization while stimulating M1 polarization, thereby helping prevent immune paralysis. Endotoxin tolerance is another significant driver of immunosuppression in sepsis [149]. Adenosine monophosphate-activated protein kinase (AMPK) has been reported to participate in the inhibition of endotoxin tolerance [150, 151]. Stimulating AMPK aids in inhibiting the production of TGF-β1 induced by LPS and its corresponding signaling pathway, enhancing the production of the proinflammatory cytokines TNF-α and IL-6 and thereby hindering the progression of endotoxin tolerance in macrophages [152]. Liu et al. discovered that AMPK activators can impede the buildup of the immunosuppressive transcription factor HIF-1α, thereby preventing the development of LPS-induced endotoxin tolerance [153]. Therefore, AMPK activators are expected to become therapeutic drugs for SALI. During the immune suppression stage of sepsis, M2 macrophage polarization contributes to immune tolerance and increases susceptibility to secondary infections through mechanisms such as IL-10 release and reduced HLA-DR expression. eCIRP activates STAT3 via IL-6R, promoting M2 polarization, which leads to excessive inhibition of M1 polarization, thereby weakening the immune response.

### Fibrosis stage

Pulmonary fibrosis is a typical manifestation of the late stage of septic lung injury and is characterized by thickening of the alveolar walls, damage to the alveolar structure, and resulting respiratory failure [154, 155]. Macrophages play a crucial role in this process, primarily because they are the main source of TGF-β [156]. TGF-β is the key effector molecule in fibrosis [157] and promotes promoting fibroblast proliferation and collagen synthesis. Macrophages can further exacerbate this process by producing growth factors such as FGF, PDGF, VEGF, and IGF-1 [158]. However, macrophages also have the ability to inhibit fibrosis by generating matrix metalloproteinases (MMPs) to degrade the extracellular matrix (ECM) [159].

Many researchers believe that pulmonary fibrosis is closely associated with a persistent inflammatory response induced by M1 polarization [160]. Under the influence of inflammation and infection, type II alveolar epithelial cells become damaged and macrophages accumulate, leading to sustained inflammation that promotes the occurrence of pulmonary fibrosis [161]. Furthermore, studies indicate that in SALI, the transcription factor IRF-5 promotes the M1 macrophage phenotype, increasing the expression of proinflammatory cytokines and high levels of iNOS [162]. Research has reported that mice deficient in IRF-5, specifically in macrophages, exhibit fibrotic responses in adipose tissue when exposed to a high-fat diet [163]. TLR2 levels are elevated in pulmonary fibrosis, and inhibiting TLR2 expression can protect mice from bleomycin-induced lung injury and fibrosis.

M2 macrophage polarization is closely associated with aberrant wound healing in fibrosis [164]. M2 macrophages reportedly aggregate at the site of injury and promote collagen synthesis in fibroblasts by releasing CCL18. Certain M2 macrophages can further increase CCL18 production by binding to type I collagen via β2 integrin and scavenger receptors, exacerbating fibrosis [165]. Research by Kral et al. revealed that in a bleomycin-induced fibrosis model, mice lacking PTEN exhibited sustained activation of PI3K, which enhanced M2 polarization of macrophages, leading to increased susceptibility to the disease [166]. Shp2 is a cytoplasmic protein tyrosine phosphatase [167], and studies have shown that Shp2 deficiency enhances JAK1/STAT6 signaling induced by IL-4, promoting M2 polarization and increasing sensitivity to pulmonary fibrosis induced by bleomycin [168]. Additionally, Ye et al. reported that IL-10 secreted by neutrophils in the bronchoalveolar lavage fluid of pulmonary fibrosis model mice may lead to M2c polarization of macrophages, which could be an important mechanism for fibrosis following ALI [169]. In the fibrosis stage, both M1 and M2 macrophage polarization contribute to tissue fibrosis. Upregulation of IRF-5 and TLR2 enhances M1 polarization, resulting in persistent inflammation that exacerbates fibrosis. M2 macrophages, driven by pathways such as PI3K and JAK1/STAT6, promote fibrosis by synthesizing collagen and secreting factors such as CCL18 and TGF-β.

## NPs regulate macrophage polarization and alleviate SALI

**Fig. 3:**
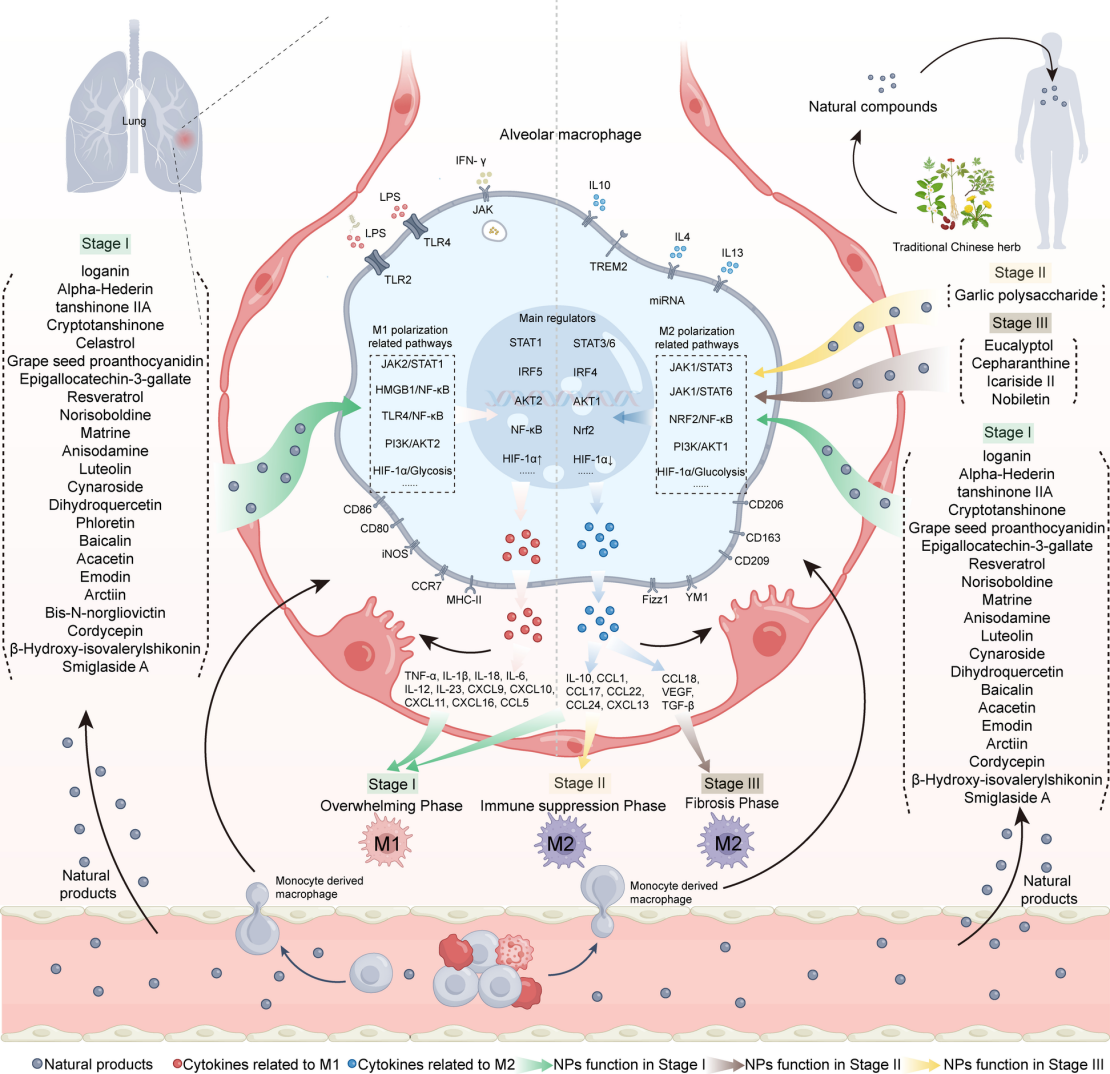
The diagram illustrates the regulatory effects of natural products on alveolar macrophage polarization during different stages of SALI progression. Alveolar macrophages, influenced by external stimuli (e.g., LPS, IFN-γ, IL-10, IL-4, IL-13), undergo M1 or M2 polarization, corresponding to the overwhelming inflammatory stage (Stage I), immune suppression stage (Stage II), and fibrosis stage (Stage III). In Stage I, natural products such as Celastrol inhibit excessive M1 macrophage polarization by targeting pathways like JAK2/STAT1, HMGB1/NF-κB, and PI3K/AKT, while alpha-Hederin not only suppresses M1 polarization but also promotes M2 polarization by regulating pathways such as JAK1/STAT3, JAK1/STAT6, and NRF2/NF-κB. In Stage II, M2 polarization dominates, leading to excessive inflammation suppression, and natural products like garlic polysaccharide modulate M2 macrophage activity. In Stage III, natural products such as eucalyptol prevent fibrosis by targeting M2 macrophage polarization pathways. Key markers and regulators of M1 and M2 macrophage polarization, including proinflammatory cytokines (e.g., TNF-α, IL-1β) and anti-inflammatory cytokines (e.g., IL-10, TGF-β), are highlighted. Green, brown, and yellow arrows represent the effects of natural products during Stages I, II, and III, respectively

NPs and their derivatives exhibit significant anti-inflammatory and tissue repair functions in preventing SALI [170–172]. Moreover, this article consolidates recent insights into the use of NPs as regulators of macrophage polarization and about their role in protecting against SALI. We provide a detailed list of these compounds and categorize them into five classes based on their chemical structure: terpenoids and terpenes, polyphenols, alkaloids, flavonoids, and others (Table 1). We also investigated the mechanism by which representative NPs act as regulators of macrophage polarization in septic lung injury, as illustrated in Fig. 3.

**Table 1:** NPs with therapeutic effects on SALI by modulating macrophage polarization (the table can be found at the end of the text.)

Compounds	Structure	PubChem CID	Molecular Formula	Dose/concentration	Model	Related pharmacological indicators	Related molecular mechanisms
Terpenoids and Terpenes							
*Loganin*		87691	C_17_H_26_O_10_	20, 40 and 80 mg/kg/day (mice); 5 μM, 10 μM, 20 μM (cell)	CLP mice) LPS (100 ng/ml) RAW264.7	CD86↓, iNOS↓, IL-1β↓, IL-18↓, TNF-α↓, IL-6↓, CD206↑, Arg-1↑	Inhibiting TLR4/NF-κB signaling; inhibits NLRP3 inflammasome activation
*Alpha-Hederin*		73296	C_41_H_66_O_12_	0.3 and 3 mg/kg/day (mice)	CLP mice	CD86↓, iNOS↓, TNF-α↓, IL-6↓, CD206↑,	Inhibiting TLR4/NF-κB activation in septic mice
*Tanshinone IIA*		164676	C_19_H_18_O_3_	10 mg/kg/day (mice)	LPS (2 mg/kg) mice	IL-6↓, IL-18↓, TNF-a↓, Ly6C↓, CD163↑	Inhibiting the TLR4/NF-κB and HIF pathways
*Cryptotanshinone*		160254	C_19_H_20_O_3_	15, 30 and 60 mg/kg/day (mice); 2.5 μM, 5 μM, 10 μM (cell)	LPS (5 mg/kg) rat; LPS (1 μg/mL) RAW264.7	CD68/CD86↓, iNOS↓, TNF-α↓, IL-1β↓, IL-6↓, Arg-1↑, IL-10↑	Inhibiting LPS-induced glycolysis
*Celastrol*		122724	C_29_H_38_O_4_	1 mg/kg/day (mice); 1 μM (cell)	CLP/LPS (1 mg/kg) mice; LPS (100 ng/ml) RAW264.7	OCR ↑, ECAR↓, TNF-α↓, IL-1β↓, IL-6↓	Inhibiting PKM2 signaling and glycolysis; inhibits the proinflammatory activity of HMGB1
*Eucalyptol*		2758	C_10_H_18_O	50 mg/kg/day (mice); 0, 5, 15, and 45 μM (cell)	BLM (1.5 U/kg) mice; 15 ng/ml IL-13 BMDM	Arg-1↓, Ym-1↓, IL-13↓, TNF-α↓, IL-6↓, TGF-β↓,	Inhibiting STAT6/KLF4 signaling pathway
Polyphenol							
*Grape seed proanthocyanidin*		108065	C_31_H_28_O_12_	25, 50 and 75 mg/kg/day (mice); 50 or 100 μg/ml (cell)	LPS (10 mg/kg) mice; 1 μg/mL LPS MH-S	CD86↓, iNOS↓, TNF-α↓, IL-1β↓, IL-6↓, CD206↑	Activating TREM2/PI3K/AKT signaling pathway
*Epigallocatechin-3-gallate*		65064	C_22_H_18_O_11_	15 mg/kg (mice)	LPS (2 mg/kg) mice	iNOS↓, TNF-α↓, IL-1β↓, COX-2↓, IL-6↓, Arg-1↑, Ym1↑	Promoting IL-4/Arg-1 signaling pathway
*Resveratrol*		445154	C_14_H_12_O_3_	40 mg/kg (mice); 50 μM (cell)	LPS (5 mg/kg) mice; LPS (500 ng/mL) BMDM	iNOS↓, TNF-α↓, CXCL15↓, IL-1β↓, IL-6↓, Arg-1↑, CD206↑	Inhibiting STAT3/SOCS3 signaling pathway
Alkaloid							
*Norisoboldine*		14539911	C_18_H_19_NO_4_	10, 20 and 40 mg/kg/day (mice); 10 μM, 20 μM, 40 μM (cell)	LPS (10 mg/kg) mice; LPS (10 µg/mL) RAW264.7	CD86↓, IL-1β↓, IL-6↓, TNF-α↓, MIP-2↓, iNOS↓, IL-12↓, CD163↑, IL-10↑, IRF4↑	Activating PKM2, and inhibits PKM2 from cytoplasm to nuclear, attenuates HIF-1α expression
*Matrine*		91466	C_15_H_24_N_2_O	100 mg/kg (mice)	CLP mice	iNOS↓, IL-6↓, TNF-α↓, IL-12↓, IL-1β↓, CD68↓, CD206↑, IL-10↑, Arg-1↑	Inhibiting the NF-κB signaling pathway and the p53-induced proapoptotic pathway
*Anisodamine*		6918612	C_17_H_23_NO_4_	1 mg/kg (mice)	LPS (20 μg/mice) mice; LPS (100 ng/mL) or IL-4 (10 ng/mL) BMDM	CD206↑, Arg-1↑, iNOS↓, IL-6↓, IL-10↑	Activating IRF4 and the NRF2/ARE pathway
*Cepharanthine*		10206	C_37_H_38_N_2_O_6_	10 mg/kg (mice);0.5 μg/ml (cell)	BLM (2 mg/kg) mice; IL-4 (20 ng/mL) RAW264.7	CD206↓, IL-6↓, TGF-β1↓, TNF-α↓, α-SMA↓	Reducing TGF-β1 expression
Flavonoid							
*Luteolin*		5280445	C_15_H_10_O_6_	0.2 mg/kg (mice)	CLP mice; LPS (30 ng/mL) or IL-10 (100 ng/mL) RAW264.7	iNOS↓, IL-1β↓, IL-6↓, TNF-α↓, IL-17A↓, CD206↑, IL-10↑	Increasing the proportion of Tregs and promotes IL-10 expression
*Cynaroside*		5280637	C_21_H_20_O_11_	5 and 10 mg/kg (mice); 10 μM (cell)	CLP (mice); LPS (1 μg/mL) RAW264.7	iNOS↓, IL-6↓, IL-1β↓, TNF-α↓, CD206↑, IL-10↑, Arg-1↑	Promoting NRF2/HO-1 signaling pathway
*Dihydroquercetin*		439533	C_15_H_12_O_7_	25 mg/kg and 50 mg/kg (mice); 1, 2.5, 5 μg/mL (cell)	LPS (10 mg/kg); LPS (5 μg/mL) RAW264.7	CD86↓, IL-1β↓, IL-6↓, TNF-α↓, iNOS↓, CD206↑, Arg-1↑, CD163↑, IRF4↑	Promoting IRF4 and the miR-132-3p/FBXW7 axis
*Phloretin*		4788	C_15_H_14_O_5_	10 mg/kg and 50 mg/kg (mice); 25 μM (cell)	LPS (5 mg/kg) mice; LPS (100 ng/mL) primary peritoneal macrophages	ECAR↓, IL-1β↓, TNF-α↓, MCP-1↓	Inhibiting GLUT1-mediated glycolysis
*Baicalin*		64982	C_21_H_18_O_11_	30 mg/kg (mice); 2.5 μM, 5 μM, 10 μM (cell)	LPS (3.75 mg/kg) mice; LPS (0.5 μg/ mL) BMDM and RAW264.7	CD86↓, IL-6↓, TNF-α↓, MCP-1↓, CD206↑, Arg-1↑	Alleviating Drp1-induced mitochondrial impairment
*Acacetin*		5280442	C_16_H_12_O_5_	80 mg/kg (mice), 30 μM, 60 μM (cell)	CLP mice; LPS (1 μg/mL) RAW264.7	iNOS↓, CD86↓, CD206↑, Arg1↑, TNF-α↓, IL-1β↓, IL-6↓	Inhibiting MAPK/NF-κB signaling pathway
*Icariside II*		44587252	C_27_H_30_O_10_	2.5,5 and 10 mg/kg/day (mice); 1.5, 2.5 and 5 µM (cell)	BLM(2.5 U/kg) mice; IL-4 (20 ng/ml) RAW264.7	CD206↓, Arg1↓	Inhibiting PI3K/Akt/β-catenin signaling pathway
*Nobiletin*		72344	C_21_H_22_O_8_	20 and 40 mg/kg/d (mice), 25 or 12.5 μM (cell)	BLM(5 U/kg) mice; IL-4 (20 ng/mL) MH-S	CD206↓, Arg1↓	Activating AMPK/mTOR signaling pathway
Others							
*Emodin*		3220	C_15_H_10_O_5_	40, 60 and 80 mg/kg/day (rat)	LPS (7.5 mg/kg) rats	CD86↓, IL-1β↓, TNF-α↓, IL-6↓, iNOS↓, CD206↑, IL-10↑, Arg-1↑	Inhibiting NF-κB signaling pathway and MAPK phosphorylation
*Arctiin*		100528	C_27_H_34_O_11_	10, 20, 40, 80 mg/kg/day (mice)	LPS (4 mg/kg) mice	CD86↓, CD206↑	Inhibiting macrophage pyroptosis
*Bis-N-norgliovictin*		13989141	C_14_H_18_N_2_O_3_S_2_	3, 10, 30 mg/kg (mice); 0.5, 1, 2 mg/ml (cell)	LPS (10 mg/kg) mice; LPS (100 ng/ml) RAW264.7	iNOS↓, CD11c↓, TNF-a↓, IL-6↓, MCP-1↓, IL-10↓, Arg-1↑	Inhibiting TLR4/NF-κB signaling pathway
*Cordycepin*		6303	C_10_H_13_N_5_O_3_	0, 5, 12.5 and 25 mg/kg/day (mice)	CLP mice	iNOS↓, Arg-1↑, CD206↑, TNF-α↓, MCP-1, IL-6↓, IL-1β↓, IL-10↑, TGF-β↑	Inhibiting NF-κB/p65 signaling pathway
*β-Hydroxy-isovalerylshikonin*		341393896	C_21_H_24_O_7_	2.5 mg/kg/day (mice); 0.5 or 1 μM (cell)	LPS (10 mg/kg) mice; LPS (100 ng/mL) RAW 264.7	CD86↓, TNF-α↓, IL-1β↓, CD206↑, Arg-1↑	Activating AMPK/ NRF2 signaling pathway
*Smiglaside A*		51255484	C_30_H_32_O_9_	3 or 10 mg/kg/d (mice); 10 μM (cell)	LPS (10 mg/kg) mice; LPS (100 ng/mL) RAW264.7 and primary mouse peritoneal macrophages	CD206↑, Arg-1↑, TNF-α↓, IL-1β↓, iNOS↓,	Activating the AMPK-PPARγ pathway

The biological activity of natural products is strongly linked to their structure. Terpenoids and terpenes, derived from isoprene units (C5H8), exhibit diverse biological effects depending on the number of isoprene units [173]. For instance, monoterpenes (e.g., *loganin*) are well known for their antimicrobial and anti-inflammatory properties, while diterpenes (e.g., *cryptotanshinone*) are widely used in anticancer therapies. Polyphenols are well-known for their antioxidant properties, primarily due to the hydroxyl groups attached to their aromatic rings. These hydroxyl groups enhance the compound's ability to donate electrons and neutralize free radicals. As a result, polyphenols are often used as potent antioxidants to prevent oxidative damage and reduce inflammation. For instance, *grape seed proanthocyanidins* are common polyphenolic compounds with notable antioxidant and anti-inflammatory effects. Research has demonstrated that they can alleviate LPS-induced inflammatory responses in RAW264.7 macrophages by inhibiting the NRF2 and NF-κB pathway [174]. Alkaloids typically contain a nitrogen atom, which is crucial for their biological activity. Many also feature aromatic rings, which improve their ability to cross lipid membranes and interact with enzymes or receptors. In the case of *matrine*, the basic nitrogen atom in its quinolizidine ring enhances its solubility and facilitates hydrogen bond formation, strengthening receptor binding [175]. Flavonoids, a subclass of polyphenols, contain two aromatic rings that are crucial for receptor binding and antioxidant activity. Additionally, many flavonoids are glycosylated, with sugar moieties that influence their solubility, bioavailability, and ability to cross cell membranes. *Baicalein* features three hydroxyl groups at positions 5, 6, and 7 on the A-ring, which are essential for its antioxidant effects by scavenging free radicals and chelating metal ions [176, 177]. Its planar, conjugated aromatic structure enhances interactions with biological membranes and enzymes, supporting its anti-inflammatory, anticancer, and antiviral properties. Furthermore, studies have shown that baicalein reduces inflammation by inhibiting the JAK/STAT1 pathway and lowering TNF-α levels.

### Terpenoids and terpenes

Terpenoids and terpenes are common in nature and possess anti-inflammatory, antioxidant, antibacterial, and macrophage polarization-modulating properties [178]. These characteristics mean they are commonly employed in managing SALI [179, 180]. Terpenoid compounds such as *loganin*, *alpha-hederin*, *tanshinone IIA*, *cryptotanshinone*, and *celastrol* have shown potential in alleviating lung injury by regulating macrophage polarization. These compounds not only reduce inflammatory responses but also promote tissue repair, suggesting they are multiple effective approaches for treating lung injury.

*Loganin*, an iridoid glycoside isolated from the fruit of *Cornus officinalis*, has been shown to have protective effects against lung injury, nonalcoholic liver disease, cardiovascular damage, renal injury, neuropsychiatric disorders, and arthritis [181–183]. In SALI, *loganin* effectively inhibits M1 macrophage polarization and reduces alveolar structure damage by inhibiting the NF-κB signaling pathway [184]. Moreover, its anti-inflammatory effects are linked to the suppression of NLRP3 inflammasome activation [185, 186]. *Loganin* has been reported to upregulate SIRT1, inhibit NF-κB-p65 acetylation, and reduce the expression of TNF-α, IL-6, and IL-1β, thereby preventing M1 polarization [187]. By inhibiting M1 polarization, *loganin* also helps prevent oxidative stress damage and T-cell dysregulation [188]. In all, *loganin* reduces pro-inflammatory cytokines and oxidative stress in SALI by inhibiting M1 macrophage polarization via the NF-κB pathway and NLRP3 inflammasome suppression.

*Alpha-hederin*, a terpenoid compound extracted from grapevines, is renowned for its diverse biological activities, including anticancer, anti-inflammatory, antitumor, and antioxidant properties [189–191]. It has been reported to increase the levels of the M2 polarization marker CD206 in the lung tissue of mice with SALI, promoting tissue repair. *Alpha-hederin* inhibits the production of the M1 polarization markers CD86 and iNOS and reduces the expression levels of proinflammatory cytokines, specifically TNF-α and IL-6, in a dose-dependent manner [192]. *Alpha-hederin* exerts its anti-inflammatory effects by targeting the NF-κB signaling pathway through mechanisms involving the STING/NF-κB and TLR4/PIP2/NF-κB/NLRP3 pathways [193, 194]. Additionally, *alpha-hederin* influences M1 macrophage polarization by reducing SIRT6-dependent glycolysis [195].

*Tanshinone IIA*, the principal lipophilic component of *Salvia divinorum*, has antioxidant, anti-inflammatory, and anti-fibrotic properties [196, 197]. In the LPS-induced ALI model, *tanshinone IIA* inhibits the activation of the NF-κB and HIF pathways, increases the proportion of M2 macrophages, and reduces the proportion of M1 macrophages [198]. In LPS-induced RAW264.7 cells, *tanshinone IIA* enhances the expression of the M2 markers CD206, IL-10, Arg1, and FIZZ1 by improving mitochondrial function and regulating the TLR4-HMGB1/CEBP-β pathway [199]. Concurrently, it reduces the expression of the M1 markers iNOS, TNF-α, and IL-1β, thereby inhibiting M1 polarization [200].

*Cryptotanshinone* is a lipophilic bioactive compound extracted from the roots of *Salvia divinorum*, that belongs to the diterpene quinone class, and it has anti-inflammatory, anticancer, antioxidant, and antifibrotic properties [201–203]. In the LPS-induced ALI model, *cryptotanshinone* activates AMPK, blocks the metabolic shift from aerobic oxidation to glycolysis induced by LPS, inhibits macrophage polarization toward the M1 phenotype, and promotes the M2 phenotype [204]. In LPS-induced RAW264.7 cells, *cryptotanshinone* inhibits the NLRP3/TGF-β1 pathway, reverses M1 macrophage polarization towards the M2 phenotype, and regulates the MMP-9/TIMP-1 balance, alleviating pulmonary fibrosis [205]. These findings indicate that *cryptotanshinone* has dual regulatory effects on macrophage polarization during lung injury.

*Celastrol*, a pentacyclic triterpenoid compound derived from the traditional Chinese herb *Tripterygium wilfordii*, has protective effects against tissue damage in conditions such as sepsis, arthritis, and brain injury [206–208]. *Celastrol* suppresses the release of the proinflammatory cytokines TNF-α, IL-1β, and IL-6; inhibits the PKM2-dependent Warburg effect; and binds to Cys106 in the HMGB1 protein, which helps to rebalance macrophage glycolytic metabolism, suppress M1 polarization, and alleviate lung damage caused by sepsis [209]. Additionally, *celastrol* activates the NRF2/HO-1 pathway, inhibits the activation of NF-κB, and reduces the expression of TNF-α, IL-6, IL-1β, and iNOS, further suppressing M1 polarization [210]. Collectively, these mechanisms contribute to its protective effect against SALI.

*Eucalyptol,* a terpene oxide derived from *eucalyptus*, is known for its anti-inflammatory and antioxidant properties [211, 212]. Studies have indicated that eucalyptol can alleviate bleomycin-induced pulmonary fibrosis, suppressing the expression of arginase-1, Ym-1, IL-13, and transforming growth factor (TGF)-β1, as well as reducing the production of IL-13, IL-6, and TNF-α, which inhibits M2 macrophage polarization [213]. Furthermore, in vitro research has found that eucalyptol can inhibit the phosphorylation of STAT6 and decrease the expression of its downstream factor KLF4, thereby suppressing M2 polarization. This suggests that eucalyptol may achieve its effects in pulmonary fibrosis damage by inhibiting M2 polarization.

### Polyphenols

Polyphenols are secondary metabolites commonly found in plants. Clinical and experimental research suggests that consuming a diet rich in polyphenols could promote the repair of lung tissue through their anti-inflammatory, antioxidant, and anti-apoptotic effects [214, 215]. A systematic review investigated the impact of *resveratrol* on circulating inflammatory markers, including IL-6, TNF-α, and high-sensitivity C-reactive protein (hs-CRP), in adults aged 17 and older, with a total of 736 participants. The results showed that polyphenol supplementation led to a significant reduction in the levels of TNF-α and hs-CRP [216]. Notably, research on the impact of polyphenols on macrophage polarization is rapidly increasing. Existing evidence suggests that specific polyphenols, such as *grape seed proanthocyanidin*, *epigallocatechin-3-gallate*, and *resveratrol*, can improve lung tissue damage by modulating macrophage polarization. These findings have significant implications for treating SALI, particularly in the context of targeting macrophage behavior to reduce inflammation and promote tissue repair.

*Grape seed proanthocyanidin* is an active compound extracted from grape seeds that can significantly modulate macrophage polarization phenotypes [174]. It reduces the expression levels of M1 markers such as iNOS and the proinflammatory cytokines TNF-α, IL-6, and IL-1β while increasing the expression of the M2 marker CD206. *Grape seed proanthocyanidin* regulates macrophage polarization from the M1 phenotype to the M2a phenotype via the TREM2/PI3K/AKT pathway [174]. Additionally, studies have shown that *grape seed proanthocyanidin* effectively exerts anti-inflammatory effects on LPS-induced RAW264.7 macrophages by inhibiting the MAPK and NF-κB pathways [217].

*Epigallocatechin-3-gallate* (EGCG) is an active ingredient extracted from green tea that plays a significant role in regulating M1/M2 polarization [218]. It inhibits the expression of inflammatory and M1 polarization markers such as iNOS, TNF-α, IL-1β, and IL-6 induced by LPS [219] while enhancing the expression of the M2 phenotype markers KLF4, Arg1, and YM1 induced by IL-4. Additionally, EGCG reduces oxidative damage and promotes lung cell regeneration. EGCG has been reported to alter macrophage polarization by inhibiting the expression of early ferroptosis and apoptosis markers induced by LPS in macrophages [220].

*Resveratrol*, a plant antioxidant derived from red grapes, is known for its anti-inflammatory properties [221]. It controls macrophage polarization through the SOCS3 pathway, reducing the expression levels of MIP-2, TNF-α, IL-1β, IL-6, IL-12, IL-33, and IL-18 [221]. Additionally, *resveratrol* increased the ratio of M2 cells to M1 cells. *Resveratrol* also inhibits inflammation by inducing macrophage pyroptosis and apoptosis while regulating macrophage polarization [222]. SIRT1 is reportedly upregulated in a *resveratrol*-mediated ALI animal model and can suppress the expression of M1 macrophages [223].

### Alkaloids

Alkaloids are a diverse group of nitrogen-containing organic compounds that are widely distributed throughout nature [224]. Numerous studies have shown that many alkaloids exhibit significant biological functions, including anti-inflammatory, oxidative stress prevention, and anti-apoptotic effects, making them widely used to treat lung injury diseases [225, 226]. Among these compounds, *norisoboldine*, *matrine*, and *anisodamine* alkaloids have the potential to regulate macrophage polarization and can be used to alleviate lung injury.

*Norisoboldine* is an aporphine alkaloid extracted from the traditional Chinese herb Linderae Radix [227]. It has been shown to regulate macrophage polarization in models of ulcerative colitis, specific dermatitis, asthma, and other conditions, aiding in reducing inflammatory damage [228, 229]. *Norisoboldine* can activate PKM2, reduce HIF-1α expression, increase PGC-1α and peroxisome proliferator-activated receptor (PPAR)-γ expression, inhibit inflammation, and promote macrophage M2 polarization [230, 231]. Additionally, *norisoboldine* promotes macrophage M2 polarization by inhibiting glycolysis and enhancing oxidative phosphorylation in RAW264.7 cells [232].

*Matrine* is an alkaloid extracted from traditional Chinese herbs [175]. It participates in regulating macrophage polarization phenotypes and has anti-apoptotic effects. *Matrine* can reverse the downregulation of SIRT1 induced by sepsis. Additionally, *matrine* deacetylates the NF-κB p65 subunit and p53, inhibiting the p53-induced pro-apoptotic pathway in septic lungs and thereby promoting M2 macrophage polarization [233]. *Matrine* also inhibits NF-κB and reduces the expression of SOCS3, thereby suppressing the release of proinflammatory cytokines and decreasing M1 polarization [234]. Studies have demonstrated that *matrine* prevents M1 polarization of macrophages induced by AGEs by inhibiting the oxidative stress-mediated TLR4/STAT1 signaling pathway activated by RAGE [235].

*Anisodamine* is an alkaloid derived from the traditional Chinese herb *Scopolia*, that exhibits anti-inflammatory and antioxidant properties [236], and has the ability to modulate macrophage polarization. Its application is quite widespread in critical illnesses such as septic shock, sepsis-induced cardiac dysfunction, and acute pancreatitis [237–239]. In an LPS-induced ALI model, *anisodamine* treatment attenuated LPS-induced lung damage by reducing the levels of IL-6, iNOS, and IL-12 in lung tissue. Moreover, it increases the protein levels of IL-10, Arg-1, and CD206, inhibits LPS-induced M1 polarization, and enhances M2 polarization [240]. *Anisodamine* regulates this process by modulating methylation, which suppresses the G9a-mediated silencing of IRF4. Additionally, *anisodamine* has been reported to activate the NRF2/ARE pathway, enhancing cellular antioxidant capacity, inhibiting cellular aging, and alleviating lung injury [241]. Furthermore, *anisodamine* has anti-apoptotic effects and regulates macrophage polarization [242].

*Cepharanthine*, a naturally occurring alkaloid and the only dibenzoquinolines alkaloid approved for human use, is derived from *Stephania Cephalantha Hayata* and possesses a variety of biological functions [243]. Studies have revealed that in a mouse model of pulmonary fibrosis, treatment with CEP can ameliorate BLM-induced lung inflammation and the lung coefficient, while also reducing the expression levels of M2 macrophage marker CD206 in lung tissue, as well as the expression levels of α-SMA, fibronectin, and collagen I[244]. This suggests that CEP may improve pulmonary fibrosis by inhibiting M2 macrophage polarization and blocking the activation of fibroblasts.

### Flavonoids

Flavonoids constitute a category of organic compounds widely present in nature and are sourced from various traditional herbs as well as fruits and vegetables [245]. Several studies have shown that flavonoids possess anti-inflammatory, antioxidant, and antiviral properties [246, 247] Flavonoids such as *luteolin*, *cynaroside*, *dihydroquercetin*, *phloretin*, *baicalin*, and *acacetin* are widely used to treat lung injury diseases because they modulate macrophage polarization. Flavonoids are characterized by poor water solubility and low oral bioavailability, with factors such as digestive enzymes altering their structure and reducing their therapeutic efficacy. Their effects are dose-dependent; for instance, luteolin alleviates NLRP3 inflammasome activation and modulates macrophage polarization in RAW264.7 cells [248], reducing inflammation at moderate doses but exacerbating it at higher doses. This dose-dependent behavior complicates the determination of optimal clinical dosages.

*Luteolin* is a flavonoid compound that can be obtained from various traditional herbs, vegetables, and fruits [249]. These studies suggest that *luteolin* possesses anti-inflammatory properties, modulates immune responses, and has the ability to prevent organ tissue damage during sepsis [250]. In a CLP-induced ALI animal model, measurements of Tregs proportions in splenic mononuclear cells and peripheral blood mononuclear cells (PBMC) revealed that treating with *luteolin* increased the proportion of Tregs and IL-10 expression while reducing the expression of iNOS and increasing the expression of CD206, which promoted M2 macrophage polarization and consequently alleviated lung injury [251]. Other studies have also indicated that *luteolin* inhibits the ICAM-1/NF-κB pathway, reducing the expression of the M1 marker iNOS and suppressing the production of IL-6, IL-1β, and TNF-α, thereby inhibiting M1 polarization [252] Additionally, *luteolin* is involved in regulating macrophage polarization pathways, including the PI3K/AKT [253], NLRP3 inflammasome [248], and cell death pathways [254]. These findings suggest that *luteolin* could be a potential natural remedy for lung injury.

*Cynaroside* is a flavonoid compound derived from traditional herbal medicine known for its various effects, including antimicrobial, antioxidant, anti-inflammatory, anticancer, and antidiabetic properties [255, 256]. Recent research has shown that the anti-inflammatory function of *cynaroside* can be used to alleviate organ damage caused by sepsis [257]. In the CLP-induced mouse model, treatment with *cynaroside* reduced the expression levels of IL-1β and TNF-α, inhibited the production of the M1 marker iNOS, and promoted the generation of the M2 marker CD206, thereby alleviating lung injury. Additionally, another study revealed that *cynaroside* promotes the formation of PKM2 tetramers by preventing their translocation to the nucleus, inhibits the phosphorylation of PKM2 at Y105, and reduces the binding of PKM2 to HIF-1α, thus inhibiting glycolysis and facilitating the transition of macrophages from the M1 phenotype to the M2 phenotype [258] Additionally, intervention with an NRF2 inhibitor further confirmed the NRF2/HO-1 pathway-dependent regulation of polarization by *cynaroside* [257]. These findings suggest that *cynaroside* is promising as a valuable natural agent for managing lung damage caused by sepsis.

*Dihydroquercetin*, a class of flavonols, has been demonstrated to possess anti-inflammatory, antioxidant, anti-tumor, and antiviral properties [259]. In an LPS-induced ALI mouse model, *dihydroquercetin* treatment has been shown to reduce the levels of IL-1β, IL-6, and TNF-α, thereby inhibiting the inflammatory response [260]. Additionally, research has shown that *dihydroquercetin* intervention can increase the expression of the M2 polarization-related factor IRF4 and activate the miR-132-3p/FBXW7 axis, promoting macrophage M2 polarization and thereby alleviating LPS-induced pulmonary edema and lung injury [63, 261, 262]. These findings indicate that *dihydroquercetin* has promising therapeutic potential for modulating immune responses and mitigating inflammatory damage.

*Phloretin* is a natural dihydrochalcone flavonoid extracted from apricots and apples that is known for its anticancer, anti-inflammatory, antioxidant, and drug toxicity properties [263, 264]. *Phloretin* has been shown to ameliorate LPS-induced ALI in both in vivo and in vitro trials. As a GLUT1 inhibitor, *phloretin* blocks glucose transport and inhibits glycolysis in a GLUT1-dependent manner [265], thereby suppressing M1 polarization and significantly ameliorating LPS-induced lung pathological damage and inflammatory responses. Moreover, metabolites derived from *phloretin*, specifically *4-O-β-d-glucuronide* and *6-methoxyl-phloretin-2-O-β-d-glucuronide*, decrease the levels of iNOS, a marker of M1 macrophage polarization, while increasing the expression of IL-10 in LPS-induced RAW264.7 cells [266]. These findings suggest that *phloretin* and its metabolites have potential therapeutic value in modulating macrophage polarization and anti-inflammatory processes.

*Baicalin*, an extract derived from the roots of *Scutellaria baicalensis*, has potent anti-inflammatory and antioxidant properties in various disease models, including influenza virus infection, heart disease, diabetes, and ulcerative colitis [176, 177]. *Baicalin* can alleviate inflammation damage by modulating macrophage polarization phenotypes. In an LPS-induced ALI mouse model, treating with *baicalin* decreased the TNF-α and IL-6 levels in bronchoalveolar lavage fluid [267]. Similarly, *baicalin* also reduces TNF-α and IL-6 levels in LPS-induced RAW264.7 cells, reflecting its inhibitory effect on M1 macrophage polarization. It has been reported that in an inflammatory environment, *baicalin* regulates macrophage polarization by suppressing the NF-κB, JAK-STAT1/4, and RhoA/ROCK pathways [268–270]. These findings indicate that *baicalin* has significant potential for anti-inflammatory effects and immune response modulation, suggesting promising prospects for treating inflammation-related diseases.

*Acacetin* is a flavonoid compound extracted from *Robinia pseudoacacia* that has been shown to have protective effects against SALI, cardiac injury, skin photoaging, and gastric cancer [271, 272]. In the ALI animal model, intervention with *acacetin* suppressed the expression of the M1 markers iNOS and CD86 and reduced the levels of proinflammatory cytokines such as TNF-α, IL-1β, and IL-6. Additionally, *acacetin* enhances the expression of CD206 and Arg1 by inhibiting TRAF6, NF-κB, and COX2 [273], thereby promoting the polarization of M2 macrophages. *Acacetin* reportedly functions in an anti-inflammatory capacity by blocking the MAPK/NF-κB pathway and NLRP3 inflammasome activation [274] as well as modulating the NRF2/HO-1 pathway [275]. These findings suggest that *acacetin* has potential therapeutic value in regulating immune responses, combating inflammation, and protecting against organ damage.

*Icariside II* (ISE II), a bioactive isoflavone extracted from the traditional Chinese medicinal herb *Epimedium*, has been demonstrated to have potential therapeutic effects on pulmonary fibrosis, diabetes, neurodegenerative diseases, and cardiovascular conditions [276, 277]. In a mice model of pulmonary fibrosis, intervention with ISE II was found to ameliorate lung inflammation and lung coefficient induced by BLM, while also reducing the expression levels of M2 macrophage marker CD206, as well as α-SMA, fibronectin, and collagen I in lung tissue [278]. Furthermore, treatment with ISE II effectively weakened the aggregation of M2 macrophages and downregulated the expression of M2 signature genes such as CD206, Arg-1, and YM-1[11]. These findings suggest that ISE II may improve pulmonary fibrosis by inhibiting the polarization of M2 macrophages and blocking the activation of fibroblasts.

*Nobiletin* (NOB), a polymethoxylated flavone derived from *Citrus Peel*, has emerged as a promising dietary therapy for the treatment of pulmonary fibrosis [279, 280]. NOB has been shown to effectively ameliorate bleomycin-induced pathological lung damage and collagen deposition in mice. Moreover, NOB can reduce the expression levels of proteins associated with M2 macrophages, such as CD206 and Arg1, and inhibit the polarization of M2 macrophages [281]. These findings suggest that NOB may possess potential therapeutic value in modulating immune responses and combating fibrosis.

### Others

*Emodin*, a typical anthraquinone derivative derived from the traditional Chinese herb *rhubarb*, exhibits anti-inflammatory, antitumor, and antiviral properties [282–284]. Research has shown that *emodin* can alleviate lung injury, inhibit hepatocellular carcinoma growth, promote diabetic wound healing, and reduce asthma airway inflammation by modulating the macrophage phenotype [285–287]. In an LPS-induced SALI mouse model, *emodin* alleviated lung inflammation and pulmonary edema by promoting the expression of the immunoregulatory neuropeptide VIP and significantly inhibited NF-κB and MAPK phosphorylation, activating the VIP/cAMP/PKA signaling pathway. This activation leads to the suppression of M1 factors such as IL-1β, TNF-α, IL-6, and iNOS in the serum while increasing the levels of M2 factors such as IL-10 and Arg-1 [288]. These mechanisms underscore *emodin*'s significant potential use in anti-inflammatory and immunomodulatory applications.

*Arctiin* is a lignan glycoside isolated from *Fructus arctii* that is known for its robust anti-inflammatory and antioxidant characteristics in various disease models, including lung injury, heart injury, neural inflammation, and diabetic nephropathy [289–291]. In the LPS-induced SALI mouse model, *arctiin* inhibits the extracellular signal-regulated kinase (ERK) pathway, leading to the suppression of the expression of the M1 proinflammatory factors IL-1β, IL-6, and TNF-α, consequently restraining M1 polarization [292]. These results indicate that *arctiin* has significant potential in regulating inflammatory responses and tissue protection.

*Bis-N-norgliovictin* is a small-molecule compound derived from *marine fungi* that exhibits significant antibacterial activity [293]. In both the LPS-induced RAW264.7 cell culture model and the mouse model, *bis-N-norgliovictin* inhibited TNF-α, IL-6, IFN-β, and MCP-1 production in a dose-depedent manner [294]. The anti-inflammatory effect was attributed to the downregulation of TLR4-triggered MyD88-dependent signaling pathways, including the NF-κB and IRF3 cascades. Moreover, *bis-N-norgliovictin* also enhances the expression of the anti-inflammatory cytokine IL-10. These findings suggest that *bis-N-norgliovictin* has anti-inflammatory effects by suppressing M1 polarization.

*Cordycepin* is an active ingredient extracted from *Cordyceps militaris* and has protective effects against inflammatory damage in various diseases, such as ALI, Alzheimer's disease, cardiac hypertrophy, specific dermatitis, asthma, kidney injury, and cancer [295–297]. In a CLP-induced mouse model, *cordycepin* intervention significantly downregulates the TNF-α and MCP-1 levels while increasing the IL-4 and IL-10 levels [298]. Further research has revealed that *cordycepin* modulates macrophage polarization from the M1 to the M2 phenotype by blocking NF-κB/p65, thereby alleviating lung injury and immune deficiency. Additionally, in ALI rats, *cordycepin* can induce the translocation of NRF2 from the cytoplasm to the nucleus, increase the expression and enzymatic activity of HO-1, reduce the release of TNF-α and IL-6, inhibit M1 polarization, and alleviate lung injury [299].

*β-Hydroxy-isovalerylshikonin* is a quinone compound extracted from *Lithospermum erythrorhizon* that promotes tumor cell apoptosis, anti-inflammation, and anti-lipid formation [300]. This compound can modulate macrophage polarization and alleviate lung damage caused by sepsis. In LPS-induced RAW264.7 macrophages, treating with *β-hydroxy-isovalerylshikonin* resulted in changes in the ratio of CD86 + M1 macrophages to CD206 + M2 macrophages, indicating a shift from the M1 type to the M2 type, which is linked to the activation of the AMPK/NRF2 pathway [301].

*Smiglaside A* is a phenylpropanoid glycoside derived from the traditional herb *Smilax riparia*. Research suggests that *smiglaside A* can increase the phosphorylation levels of PPARγ and AMPK, facilitating the transition of alveolar macrophages into the M2 phenotype and thereby increasing the survival rate of SALI model mice [302]. These findings suggest that *smiglaside A* might have potential therapeutic value in regulating lung immune responses and enhancing resistance.

*Garlic polysaccharide* (GP), a polysaccharide compound extracted from *garlic*, possesses a variety of biological activities including antioxidant, anti-inflammatory, lipid-lowering, and immunomodulatory effects [303, 304]. In vitro experimental research has found that GP can enhance the immune response of RAW264.7 cells, stimulate the elevation of NO, TNF-α, and IL-6 levels, promote the polarization of macrophages towards the M1 phenotype, and simultaneously inhibit the shift towards the M2 phenotype [304]. In vivo experiments using immunosuppressed mouse models have revealed that GP exhibits a dose-dependent upregulation of factors suppressed by cyclophosphamide (such as IFN-γ and IL-6), as well as levels of immunoglobulins (such as IgA and IgG) [305]. These findings suggest that GP can promote the polarization of M1 macrophages, suppress M2 polarization, and thereby facilitate the immune recovery in immunosuppressed mice.

## Conclusion and discussion

The most notable characteristic of SALI is its persistent inflammatory response, with immune system imbalance prevailing throughout the disease process. Macrophages play a pivotal role in SALI as key regulators of the innate immune system. An imbalance between proinflammatory M1 and anti-inflammatory M2 macrophages is a key factor in disease development. Correcting this M1/M2 ratio imbalance to improve SALI represents a novel therapeutic approach, which may offer more opportunities for treating SALI. The present article provides a review of NPs that have shown therapeutic effects against SALI induced by macrophage phenotype imbalance, along with their associated pharmacological pathways. This article offers researchers some profound insights: As is well known, SALI is a type of pulmonary inflammatory disease, hence anti-inflammatory therapies, including pharmaconutrients, antioxidants, corticosteroids, ibuprofen, and protease inhibitors, have been extensively studied. Additionally, cell therapies involving growth factors, colony-stimulating factors, and stem cells have garnered significant attention from researchers. Despite a plethora of intervention measures, drugs currently available for the management of SALI have not yet shown promising results. The reasons may include drug resistance, gastrointestinal discomfort, complex repeated dosing regimens, and a substantial increase in drug costs [306].

As phytopharmacological research continues to advance, an increasing number of natural medicines are demonstrating significant potential in treating SALI [307]. These natural compounds offer several therapeutic advantages: (1) The complex structures and diversity of natural compounds endow them with a rich array of biological activities; (2) Natural products possess specific biological functions within organisms, providing them with an inherent advantage in terms of drug-like properties; (3) These plant-derived compounds are relatively safe and effective, with low resistance, fewer side effects, and economic benefits. Therefore, incorporating natural medicines to explore potential alternative therapies for SALI could improve the overall survival of SALI patients. However, these natural products also have limitations in clinical and translational applications: (1) Bioavailability: The poor stability and solubility of active ingredients in natural medicines significantly limit their bioavailability, with current research largely focused on pharmacodynamics while lacking systematic pharmacokinetic studies. This challenge can be addressed by developing innovative drug delivery systems, such as nanoparticles, liposomes, and hydrogels, to enhance solubility, stability, and bioavailability. Moreover, synthetic biology and metabolic engineering can modify the activity and structure of natural products, further improving their bioavailability. (2) Safety: Natural products research faces safety challenges due to limited toxicological studies, small clinical trial sample sizes, and a lack of standardized quality control and dosage guidelines. Comprehensive toxicity assessments using high-throughput screening, animal models, and bioinformatics are essential to address risks from active components and metabolites. Robust safety validation requires multicenter randomized controlled trials. Additionally, variability in NP composition highlights the need for standardized quality control and dose escalation studies to ensure consistent efficacy and safety. (3) Scalability: Natural products face significant challenges in scalability due to low extraction efficiency and the limited availability of raw materials. To overcome these obstacles, advanced extraction technologies, such as nano-extraction and supercritical fluid extraction, can be employed to enhance extraction efficiency. Additionally, genetic engineering and synthetic biology can be leveraged to scale up production through fermentation technologies, ensuring a reliable and consistent supply of active compounds. This review summarizes the pharmacological actions and potential mechanisms of natural compounds, laying the foundation for a deeper understanding of the regulatory effects of natural compounds on macrophage polarization in SALI, which is of great significance for future drug development in SALI.

## Data Availability

Not applicable.

## Abbreviations

AIM2: Absent in melanoma 2
AKT: Protein kinase B
ALI: Acute lung injury
AMPK: Adenosine monophosphate-activated protein kinase
ARDS: Acute respiratory distress syndrome
Arg-1: Arginase-1
Brg-1: Brahma-related gene 1
CLP: Cecal ligation and puncture
COX-2: Cyclooxygenase-2
eCIRP: Extracellular cold-inducible RNA-binding protein
ECM: Extracellular matrix
EGCG: *epigallocatechin-3-gallate*
ERK: Extracellular signal-regulated kinases
HIF-1: Hypoxia-inducible factor-1
HMGB1: High-mobility group protein box 1
HO-1: Heme oxygenase 1
IFN-γ: Interferon-gamma
IFNs: Type I interferons
IL-1β: Interleukin-1 beta
IL-4: Interleukin-4
IL-6: Interleukin-6
iNOS: Inducible nitric oxide synthase
IRF3: Interferon regulatory factor 3
JAKs: Janus kinase
LPS: Lipopolysaccharide
MCP-1: Monocyte chemoattractant protein-1
MIP-2: Macrophage inflammatory protein-2
MMPs: Matrix metalloproteinases
NO: Nitric oxide
NPs: Natural products
PDGF: Platelet-derived growth factor
PI3K: Phosphoinositide 3-kinase
PIP2: Phosphatidylinositol-4,5-bisphosphate
PIP3: Phosphatidylinositol-3,4,5-trisphosphate
PK: Pyruvate kinase
PMN: Polymorphonuclear neutrophils
PPAR: Peroxisome proliferator-activated receptor
ROS: Reactive oxygen species
SALI: Sepsis-induced acute lung injury
SENP3: SUMO-specific peptidase 3
SETD2: SET domain-containing 2
SOCS: Suppressor of cytokine signaling
STATs: Signal transducers and activators of transcription
TGF-β: Transforming growth factor-beta
Tim-3: T cell immunoglobulin and mucin domain-containing protein 3
TIR: Toll/interleukin-1 receptor
TLR4: Toll-like receptor 4
TNF-α: Tumor necrosis factor-alpha
TREM2: Triggering receptor expressed on myeloid cells 2
